# The Contribution of Various MRI Parameters to Clinical and Cognitive Disability in Multiple Sclerosis

**DOI:** 10.3389/fneur.2018.01172

**Published:** 2019-01-23

**Authors:** Eszter Tóth, Péter Faragó, András Király, Nikoletta Szabó, Dániel Veréb, Krisztián Kocsis, Bálint Kincses, Dániel Sandi, Krisztina Bencsik, László Vécsei, Zsigmond Tamás Kincses

**Affiliations:** ^1^Department of Neurology, Albert Szent-Györgyi Clinical Centre, University of Szeged, Szeged, Hungary; ^2^MTA-SZTE Neuroscience Research Group, University of Szeged, Szeged, Hungary; ^3^Department of Radiology, Albert Szent-Györgyi Clinical Centre, University of Szeged, Szeged, Hungary

**Keywords:** multiple sclerosis, BICAMS, atrophy, demyelination, cognition

## Abstract

Next to the disseminated clinical symptoms, cognitive dysfunctions are common features of multiple sclerosis (MS). Over the recent years several different MRI measures became available representing the various features of the pathology, but the contribution to various clinical and cognitive functions is not yet fully understood. In this multiparametric MRI study we set out to identify the set of parameters that best predict the clinical and cognitive disability in MS. High resolution T1 weighted structural and high angular resolution diffusion MRI images were measured in 53 patients with relapsing remitting MS and 53 healthy controls. Clinical disability was inflicted by EDSS and cognitive functions were evaluated with the BICAMS tests. The contribution of lesion load, partial brain, white matter, gray matter and subcortical volumes as well as the diffusion parameters in the area of the lesions and the normal appearing white matter were examined by model free, partial least square (PLS) approach. Significance of the predictors was tested with Variable Importance in the Projection (VIP) score and 1 was used for threshold of significance. The PLS analysis indicated that the axial diffusivity of the NAWM contributed the most to the clinical disability (VIP score: 1.979). For the visuo-spatial working memory the most critical contributor was the size of the bilateral hippocampi (VIP scores: 1.183 and 1.2 left and right respectively). For the verbal memory the best predictors were the size of the right hippocampus (VIP score: 1.972), lesion load (VIP score: 1.274) and the partial brain volume (VIP score: 1.119). In case of the information processing speed the most significant contribution was from the diffusion parameters (fractional anisotropy, mean and radial diffusivity, VIP scores: 1.615, 1.321 respectively) of the normal appearing white matter. Our results indicate that various MRI measurable factors of MS pathology contribute differently to clinical and cognitive disability. These results point out the importance of the volumetry of the subcortical structures and the diffusion measures of the white matter in understanding the disability progression.

## Introduction

Multiple sclerosis (MS) is an inflammatory, demyelinating disease, which affects the central nervous system. Next to the disseminated clinical signs cognitive impairments are frequent symptoms, it can occur in 40–70% of the patients. Most commonly it affects the information processing speed, the episodic memory, the executive functions, and the visuospatial abilities. There are several psychometric surveys available, but the Brief International Cognitive Assessment for MS (BICAMS) allows a reliable, fast evaluation of the most frequently affected cognitive domains (1–3).

One of the most prominent feature of the disease is the white matter lesions identified on the MRI. The importance of these lesions is unquestionable and hence became cornerstone of the diagnosis (4) and the follow-up of therapeutic efficacy (5). However, the correlation of T2 lesion burden with clinical and cognitive impairment are modest at most, known as the clinio-radiological paradox (6, 7). Recently, increasing interest is shown about the gray matter (GM) atrophy, which has become an approach to follow-up of the therapeutic effectiveness (8, 9). The fact that it correlates stronger with the clinico-cognitive functioning gives the real importance of the GM (10, 11). While lesions and gray matter atrophy are non-specific to the underlying pathology, there are novel methods which better approximate the pathological processes. One of those is diffusion tensor imaging, which non-invasively depicts the diffusion of water in biological tissues. The molecular diffusion is blocked by cellular elements (primarily membranes). This way the diffusion profile of the water depicts the microscopic components of the tissue architecture. It is important to notice, that the axon loss and the demyelination alters the diffusion profile differently. While the axon damage is demonstrated by the alterations in axial diffusivity, the changes of radial diffusivity allude to myelin damage. Diffusion tensor imaging, with appropriate parameters was able to detect widespread alterations of the white matter, even in the non-lesioned, normal appearing white matter (NAWM) (12). These alterations were also correlating with various clinical and cognitive functions (13, 14).

Several studies investigated the correlation between various MRI markers and clinical and cognitive dysfunction (14–17), but only a few study investigated the relative importance of these MRI parameters (18–20). Despite the undisputed merit these studies have limitations, as in some of the studies deployed only low number of diffusion directions, only some of the diffusion parameters were used, others did not include all of the subcortical structures separately in the analysis and cognitive domains were evaluated separately only by a few of the investigations.

Moreover, MRI parameters are highly related and that relationship is not trivial (12), conventional linear regression analysis could not unambiguously predict the importance of the variables. The model-free partial least square (PLS) approach, besides handling the problem of collinearity, is able to distinguish a pattern of those parameters that best predicts the variable in question. In the current investigation we set out to identify those MRI parameters, which could predict the clinical disability and various domains of cognitive dysfunction with the model free PLS approach.

## Materials and Methods

### Subjects

The study was carried on 53 patients with relapsing-remitting MS diagnosis and 53 healthy, age-matched controls without history of any neurological or psychiatric diseases. Patients were enrolled from the Multiple Sclerosis Outpatient Clinic at the Department of Neurology. The diagnosis was founded on the 2005 revision of the McDonald criteria (21). The clinical disability was measured on the Kurtzke expanded disability status scale (EDSS) (22). The cognitive performance of patients was measured by Brief international assessment for MS (BICAMS). All patients were on disease-modifying therapy (Table 1). All of our patients were in a stable clinical condition, without relapses or EDSS progression in 6 months before or after the MR scans.

**Table 1:** Demographic data of the subjects.

	**Healthy**	**Patients**
n	53	53
Age (years; mean ± SD)	36.06 ± 11.06	44.34 ± 11.51
Sex (male)	16	17
Education (year ± SD)	–	13,71 ± 2,4
Disease duration (years; mean ± SD)	–	13.89 ± 9.02
EDSS score	–	1.89 ± 1.65
Therapy	–	Interferon beta: 33 glatiramer acetate: 20
Duration of the therapy (years; mean ± SD)	–	Interferon beta: 3.89 ± 3.49 glatiramer acetate: 4.0 ± 3,79

The study was approved by the National Institute of Pharmacy and Nutrition and the Regional Human Biomedical Research Ethics Committee (Ref. No.: 000002/2016/OTIG). All study participant gave their written informed contribution in accordance with the Declaration of Helsinki.

### Cognitive Assessment of the Patients

The Brief International Cognitive Assessment for MS (BICAMS) test is a short form that is a fast, sensitive and specific tool for the determination of the cognitive disability of the patient. The BICAMS test involves 3 separate tests: the symbol digit modalities test (SDMT), the first five recall trials of the California verbal learning test II. (CVLT-II) and the first three recall trials of the brief visuospatial memory test revised (BVMT-R) (23).

In our study we used the validated Hungarian version of the BICAMS test [for details of the validation process see: (2)]. For all subtests of BICAMS the patient's results were compared to the age matched control group of healthy from our earlier validation study (2) more than two standard deviation difference compared to the control database was considered as abnormal.

### Image Acquisition

MR imaging were carried out on a 1.5T GE Signa Excite HDxt MR scanner. 3D spoiled gradient echo (FSPGR: TE: 4.1 ms, TR: 10.276 ms, matrix: 256 × 256, FOV: 25 × 25 cm, Flip angle: 15 degrees, in-plane resolution: 1 × 1 mm, slice thickness: 1 mm), FLAIR (TE: 133 ms, TR: 6000 ms, TI: 1848 ms, matrix: 256 × 256, FOV: 25 × 25 cm, Flip angle: 90 degrees, in-plane resolution: 1 × 1 mm, slice thickness: 1 mm) and 60 direction diffusion-weighted images with 6 non-diffusion-weighted reference volumes (TE: 93.8 ms, TR: 16,000 ms, matrix: 96 × 96, FOV: 23 × 23 cm, Flip angle: 90 degrees, in-plane resolution: 2.4 × 2.4 mm slice thickness: 2.4 mm, b: 1,000 s/mm^2^, NEX: 2, ASSET) were acquired for all subjects.

### Evaluation of Lesion Load

Lesions were manually segmented on the FLAIR images by ET, and rechecked by ZTK having considerable experience in MS neuroradiology.

### Evaluation of Global Atrophy

The partial brain volume (PBV) was calculated with SIENAX (24), part of FSL (25, 26). SIENAX started by extracting brain and skull images from the single whole-head input data (24). Tissue-type segmentation was then carried out (27) in order to calculate the partial volume of brain, the GM and WM.

### Volumetric Analysis of the Subcortical Structures

Image analysis was carried out using tools of FSL (FMRIB Software Library, http://www.fmrib.ox.ac.uk/fsl) (25). To automatically segment the subcortical structures (28), FIRST, a deformable-model-based segmentation/registration tool was used that uses a Bayesian Appearance Model (FMRIB's Integrated Registration Segmentation Toolkit). For the automatic segmentation of structures, shape and intensity variations of subcortical structures were constructed from a training set of 336 images. With preservation of the cross-subject vertex correspondence, surface meshes were obtained with a deformable model. At each vertex a sample was taken from the normalized intensities along the surface normal. Then the vertex location and intensity variation were modeled as a multivariate Gaussian distribution. Finally, maximizing the posterior probability of the shape given the observed intensities, this model was fit to new images (10, 29). The result of the segmentation was manually checked and corrected if necessary by the first author. The volume of the segmented subcortical structures were normalized to the head size.

### Microstructural Alterations of the White Matter

Diffusion data were corrected for Eddy currents and movement artifacts by 12 degree-of-freedom affine linear registration to the first non-diffusion-weighted reference image. Diffusion images were processed by using FDT (FMRIB's Diffusion Toolbox part of FSL: www.fmrib.ox.ac.uk/fsl/fdt/). Fractional anisotropy, mean diffusivity [(λ_1_ + λ_2_ + λ_3_)/3], axial diffusivity (λ_1_) and radial diffusivity [(λ_2_ + λ_3_)/2] to the principal diffusion direction were computed for the whole brain.

The Tract-Based Spatial Statistics (TBSS) method was used to reduce possible errors resulting from misalignment of the images: A non-linear registration tool (FNIRT), which uses a b-spline representation of the registration warp field, aligned all fractional anisotropy images to a 1 × 1 × 1 mm FMRIB58_FA standard space. The data on all patients were brought into the standard space, and the mean fractional anisotropy image was created and then fed into the fractional anisotropy skeletonization program, thresholded at fractional anisotropy = 0.2 to create a mean fractional anisotropy skeleton that represented the centers of all tracts common to the group. The aligned fractional anisotropy data on each subject were then projected onto this skeleton, which resulted in the 4D skeletonized fractional anisotropy image.

A voxel-wise alteration of the diffusion parameters, (microstructural integrity index—MII), was calculated for each patient (12), by comparing the value of every voxel with the distribution from the normal subjects in the spatially matching voxel (z-score). To identify a global white matter damage we calculate the averages of these z-scores:

$$\begin{array}{l} \bar{X_{n}}=\frac{\overset{q}{\underset{i=1}{\sum }}X_{n,i}}{q},\\ \delta_{n}=\frac{\overset{q}{\underset{i=1}{\sum }}\left(\bar{X_{n}}-X_{n,i}\right)}{q},\\ Z_{X,n,j}=\frac{X_{n,j}-X_{n}}{\delta_{n}}, \end{array}$$

where *X* is the measured diffusion parameter (fractional anisotropy, mean diffusivity, axial diffusivity, and radial diffusivity) in the n^th^ voxel in the skeleton. Indices *i* and *j* are for controls and patients, respectively.

The average diffusion parameters were calculated for each patient in the lesions and normal appearing white matter: The lesions were projected to the fractional anisotropy skeleton. The manually segmented lesions were brought to the diffusion data space with 6 degree-of-freedom linear registration. Through use of the warp field and the skeleton projections of the TBSS analysis of the fractional anisotropy images, the lesion mask was brought to the skeleton with the FSL *tbss_non_fa* algorithm. The mask was finally thresholded at 0.5 and binarized to avoid any size increment arising from the interpolation.

### Analysis of the Connection Between the MR Parameters and the Clinical and Cognitive Status

**Figure 1:**
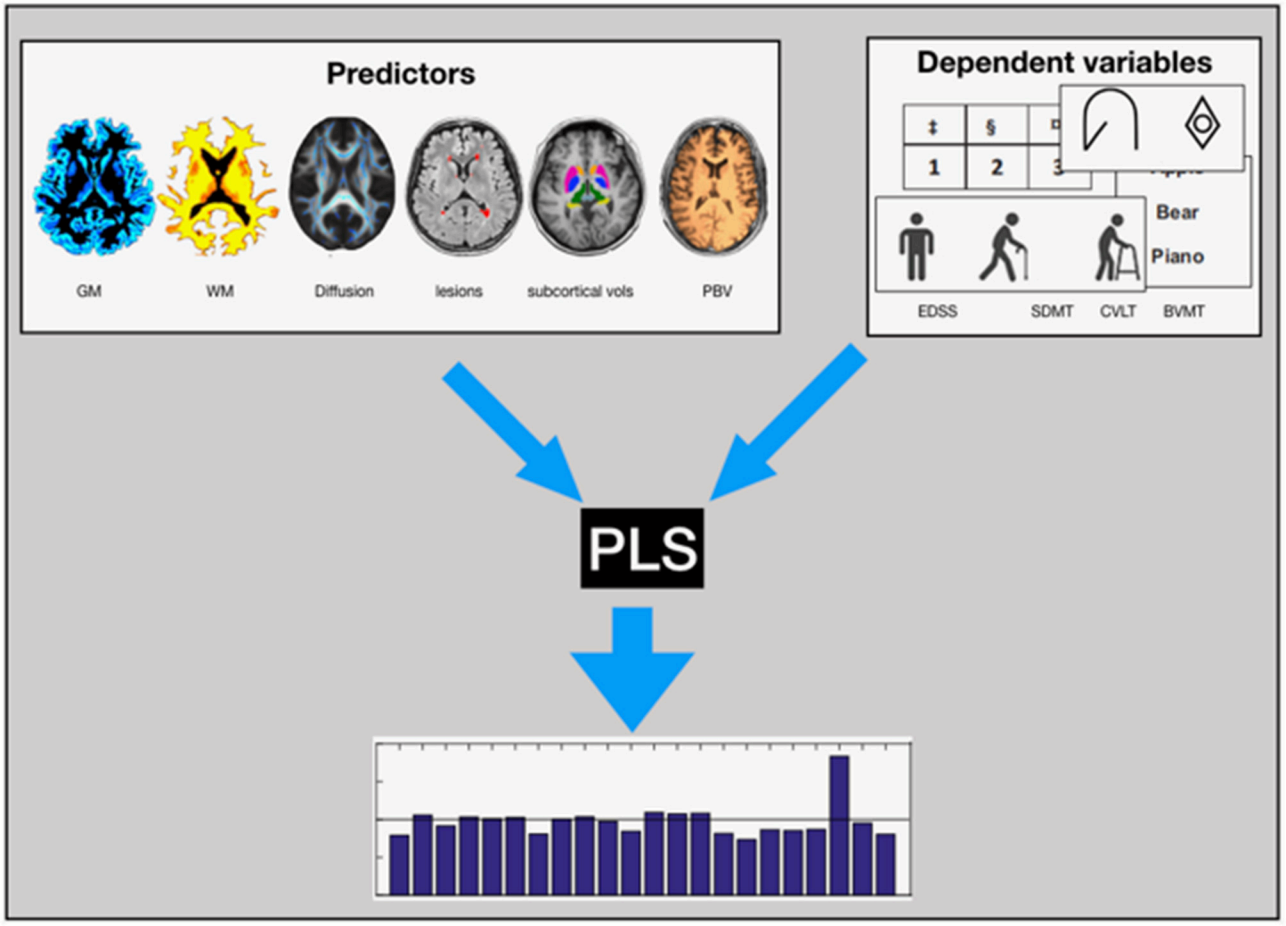
Graphical Presesntation: We used PLS regression analysis to estimate the contributions of the various MRI parameters (GM, WM, the diffusion parameters of the lesions and the normal appearing white matter, the Lesion Load, volume of the subcortical structures, and the PBV) to the EDSS and the subscores of the BICAMS test.

We used partial least square (PLS) regression analysis to estimate the contributions of the various MRI parameters (partial brain volume, normalized gray matter and white matter volume, volume of the subcortical structures, lesion load and the diffusion parameters of the lesions and the normal appearing white matter) to the EDSS and the subscores of the BICAMS test (Figure 1). If Y is an *n* × *q* matrix of dependent variables over n observations and X is an *n* × *p* matrix of predictors, PLS successively extracts latent variables (factors and loadings) from X and Y in such a way that covariance between the factors and loadings is maximized. With this approach, PLS reduces the dimensionality of the data by providing a weighted linear combination of X variables to form orthogonal components that predicts the dependent variable. In mathematical terms, Partial least squares is a linear decomposition of X and Y such that

$$\begin{array}{l} \text{X}=\text{T}{\text{P}}^{\text{T}}+\text{E},\\ \text{Y}=\text{U}{\text{Q}}^{\text{T}}+\text{F} \end{array}$$

and the covariance between T and U is maximum (30). In the above equations, T is the *n* × *r X* scores, *U* is the *n* × *r Y* scores, *P* is the *p* × *r X* loadings, *Q* is the 1 × *r Y* loadings, E, and F are residuals, and r is the number of extracted latent variables. The statistical inference on the significance of the latent variable was carried out by permutation tests on the singular values of the decomposition. The elements of the dependent variable matrix were randomly permuted 5,000 times and the singular value was recalculated to depict a null distribution. The summary of the importance for the X loadings was calculated by a Variable Importance in the Projection (VIP) score (31). Since the average of squared Variable Importance in the Projection scores is equal to 1, the “greater than one” rule was used for the selection of the important variables.

## Results

### Clinical, Cognitive, and Imaging Parameters of the Patients

The patients recruited in our study have mild to moderate disability as measured by EDSS in spite of the relatively long disease duration (Table 1). Out of the 53 patients 18 had cognitive dysfunction on one cognitive test (CVLT: 0, BVMT: 7, SDMT: 9), 8 on two tests (SDMT and BVMT: 7, BVMT and CVLT: 1) and 5 on all three tests.

For the measured MRI parameters (partial brain volume, GM and WM volume, volume of the subcortical structures and the diffusion parameters of the white matter) see Table 2.

**Table 2:** MRI parameters and cognitive scores of the subjects.

		**Multiple sclerosis**	**Healthy**	***p*-value**
Subcortical Structure volume mm^3^ (mean ± SD)	Left amygdala	1717.22 ± 304.80	1755.28 ± 235.315	0.473
	Left caudatus	4324.02 ± 611.32	4683.56 ± 561.583	0.02
	Left hippocampus	5019.21 ± 608.16	5434.64 ± 690.22	0.001
	Left pallidum	2303.76 ± 328.13	2383.04 ± 187.67	0.130
	Left putamen	6270.55 ± 749.85	6802.61 ± 601.27	0.000106
	Left thalamus	965.38 ± 1092.743	10886.14 ± 837.47	0.00000
	Right amygdale	1666.32 ± 264.20	1713.80 ± 311.28	0.399
	Right caudatus	4414.96 ± 705.49	4905.77 ± 582.28	0.000167
	Right hippocampus	5148.92 ± 551.24	5340.47 ± 663.50	0.109
	Right pallidum	2331.54 ± 250.36	2435.00 ± 181.80	0.017
	Right putamen	6248.99 ± 765.47	6658.91 ± 628.22	0.003
	Right thalamus	9380.61 ± 1092.52	10521.75 ± 925.85	0.00000
Global atrophy (mean ± SD)	Normalized PBV	1423745.72 ± 7726.26	1492340.43 ± 65602.53	0.332
	Normalized GM volume	784317.27 ± 51061.79	824838.22 ± 44204.64	0.00003
	Normalized WM volume	639428.45 ± 43973.57	667502.21 ± 36040.49	0.000498
	Normalized pGM	607231.49 ± 43383.91	642563.77 ± 35221.91	0.000012
	Normalized VCSF	48551.20 ± 20524.23	33269.52 ± 11904.28	0.000008
Diffusion parameters % (mean ± SD)	NAWM_FA	94.46 ± 5.78	–	–
	Lesioned_FA	89.90 ± 14.36	–	–
	NAWM_AD	100.85 ± 2.41	–	–
	Lesioned_AD	105.66 ± 10.07	–	–
	NAWM_MD	103.84 ± 5.14	–	–
	Lesoined_MD	112.83 ± 18.62	–	–
	NAWM_RD	108.06 ± 9.83	–	–
	Lesioned_RD	123.63 ± 35.29	–	–
Lesions	LL (mean ± SD)	9698.33 ± 9754.94	–	–
Cognitive scores (mean ± SD)	BVMT z-score	−0.67 ± 1.49	–	–
	SDMT z-score	−1.00 ± 1.22	–	–
	CVLT z-score	0.28 ± 0.98	–	–

### The Imaging Parameters Influencing Clinical Disability

**Figure 2:**
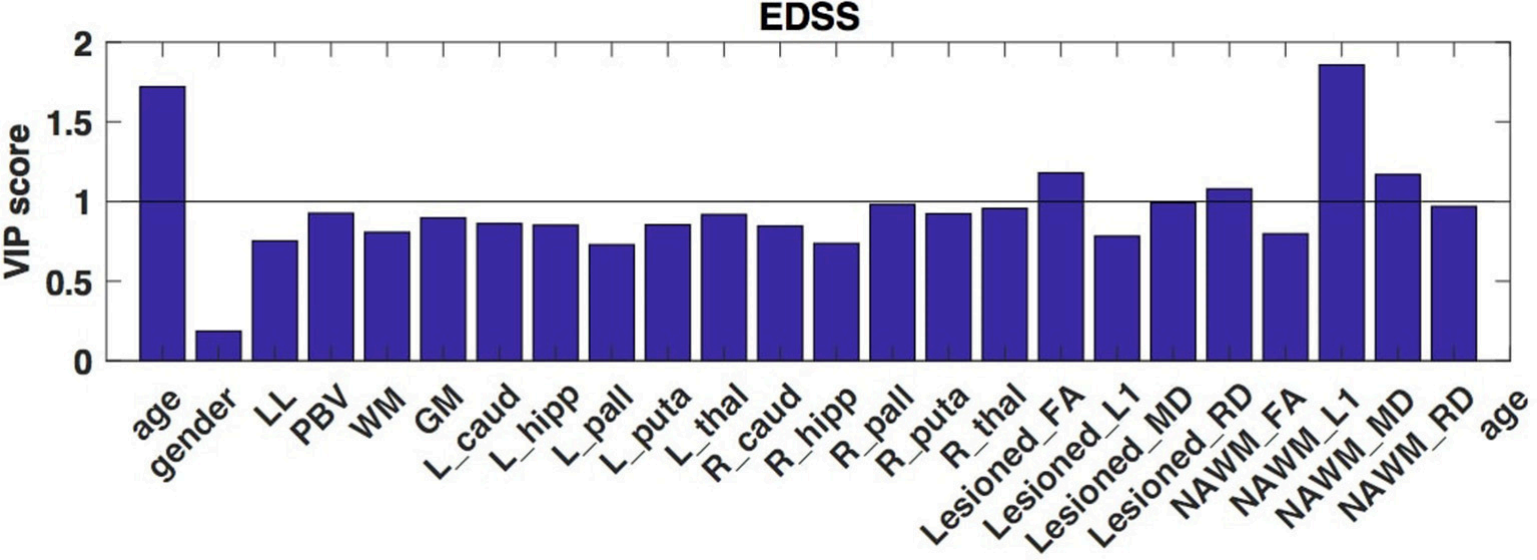
VIP scores of the partial least squares analysis that depict the optimal contrast of the independent variables predicting the clinical disability (EDSS). These VIP scores suggest, that principally the axial diffusivity of the NAWM drives the EDSS. Higher than 1 VIP scores are identified as significant.

In the first PLS analysis, the dependent variable was the EDSS. As the second latent variable interpreted only a small part of the variance of the dependent measure (<10%) and the permutation test indicated a non-significant latent variable, only the first latent variable was evaluated. The permutation test showed that the first latent variable was significant (*p* < 0.001) and responsible for 50.67% of the variation of the dependent variable and 27.08% of the predictors. The *X* loadings and the corresponding VIP scores indicated that age (VIP score: 1.72) and the axial diffusivity of the NAWM contributed the most to the clinical disability (VIP score: 1.979) (Figure 2). While far less, but still significant contributor was from the mean diffusivity of the NAWM (VIP score: 1.169), the demyelination features of the lesions (VIP_FA_: 1.17, VIP_RD_: 1.08).

### The Imaging Parameters Influencing Cognitive Functions

In these analyses the raw scores from the three subtest of the BICAMS test were used as dependent variables. The first latent variable was evaluated, as the second latent variable was responsible for just a small fraction of the variance of the dependent measure (<5% in case of BVMT and CVLT and 10% in case of SDMT) and the permutation tests indicated non-significant latent variables. The permutation test indicated that the first latent variable was significant (*p* < 0.001 for each subtests) and explained for 50.99% of the variation of the dependent variable and 23.89% of the predictors in case of the BVMT, for 50.93% of the variation of the dependent variable and 22.24% of the predictors in case of the CVLT, for 50.67% of the variation of the dependent variable and 22.43% of the predictors in case of the SDMT.

Age contributed significantly to all cognitive tests (VIP score: 1.538, 1.127, and 1.296 for BVMT, CVLT, and SDMT, respectively). Gender was significant contributor to CVLT and SDMT (VIP score: 1.356 and 1.345, respectively).

**Figure 3:**
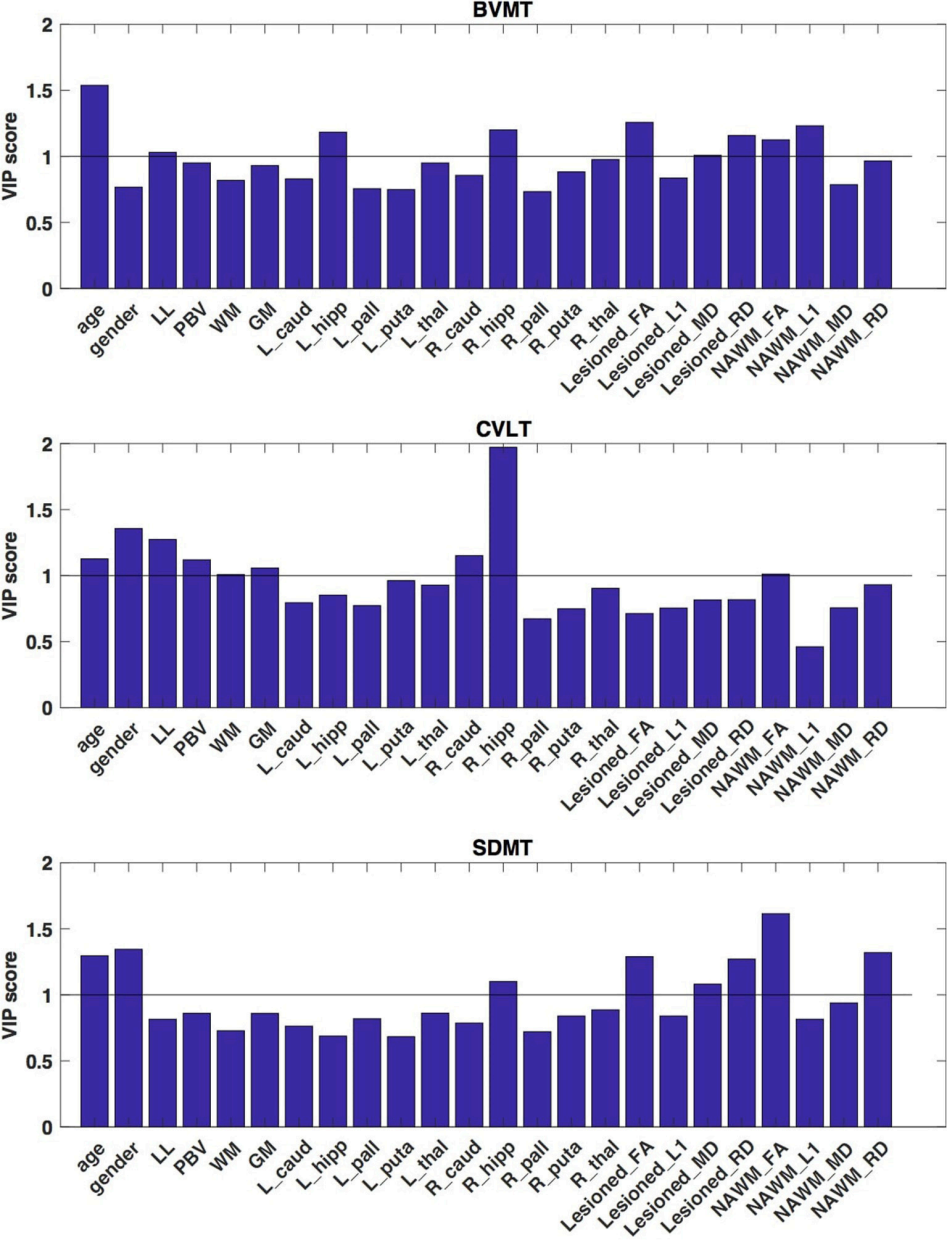
VIP scores of the partial least squares analysis that depict the optimal contrast of the independent variables predicting the cognitive functions. These VIP scores suggest, that for the BVMT the most critical contributor was the size of the bilateral hippocampi, for the CVLT the best predictor was the size of the right hippocampus and the total gray matter volume and in case of the SDMT test the most significant contribution was from the diffusion parameters fractional anisotropy, mean and radial diffusivity of the NAWM. Higher than 1 VIP scores are identified as significant.

As regarding the *visuo-spatial working memory*, the most critical contributor was the size of the bilateral hippocampi (VIP scores: 1.183 and 1.2 left and right, respectively) and the demyelination features of the lesions (VIP_FA_ score: 1.257, VIP_MD_ score: 1.008, VIP_RD_ score: 1.158) and the axon loss diffusion features of NAWM (VIP_FA_ score: 1.125, VIP_L1_ score: 1.232) (Figure 3). Lesion load was also a marginally significant contributor (VIP score: 1.031).

For the *verbal memory* the best predictor was the size of the right hippocampus (VIP score: 1.972), the lesion load (VIP score: 1.274), the partial brain volume (VIP score: 1.119) the total white matter volume (VIP score: 1.008), total gray matter volume (VIP score: 1.058), the size of the right caudate (VIP score: 1.152), and the FA of the NAWM (VIP score: 1.012) (Figure 3).

In case of the *SDMT test* the most significant contribution was from the demyelination-like diffusion parameters of the NAWM (fractional anisotropy and radial diffusivity VIP scores: 1.615, 321, respectively) (Figure 3). The FA, mean and the radial diffusivity of the lesions (VIP scores: 1.289, 1.082, and 1.271, respectively) and the size of the right hippocampus (VIP score: 1.101) also contributed significantly to the performance.

## Discussion

Our model-free analysis of multiparametric MRI data of MS patients revealed complex interplay between the clinical and cognitive disability and various MRI parameters. Clinical disability was best predicted by the axial diffusivity of the NAWM. Cognitive functions were influenced by several factors in the various domains: (i) the most critical MR contributor to the visuo-spatial working memory was the size of the bilateral hippocampi and the demyelination-like diffusion profile of the lesions, and the axon loss of the NAWM; (ii) the best predictor of the verbal memory was the size of the right hippocampus and the brain, gray and white matter volumes, and (iii) information processing speed as measured on the SDMT test was best predicted by the demyelination like diffusion parameter alterations of the white matter.

It is long known that correlation between clinical disability and lesion load is weak (6, 7). The correlation of disability with brain atrophy and primarily gray matter atrophy seems to be stronger (10, 32–36), however several voxel-wise morphometry studies failed to find correlation of focal gray matter atrophy and disability (37–42). There are conflicting results on the correlation between diffusion metrics and disability. Griffin in a ROI analysis found neither abnormal diffusion parameters in the NAWM nor correlation with disability (43). Filippi et al. (44) found modest correlation between disability and the mean diffusivity of lesions. Hasan found no connection between the fractional anisotropy of the corpus callosum and the disability of patients (45). The anisotropy of the pyramidal tract was correlating with the EDSS in 25 relapsing-remitting MS patients (46). In a whole brain TBSS analysis Onu et al. found widespread differences between controls and MS patients and within these group differences FA correlated with EDSS, hand function and ambulation (47). Ciccarelli and colleagues found connection of the clinical disability with the FA of the supra and infratentorial NAWM. In particularly the FA and MD of the cerebral peduncles showed a negative correlation with the clinical disability, measured by EDSS (48). Importantly, most of the above mentioned studies limited the analysis to FA. Also the extent of microstructural damage reported was usually far less. Previous studies have demonstrated that the radial and axial diffusivity alterations indicate demyelination and axonal pathology, respectively (49–53). In our previous analysis we showed that the demyeliniation like diffusion parameter changes can be detected in most of investigated white matter skeleton if high number of diffusion directions are investigated (12). Interestingly, our current analysis pointed out, that the clinical disability was best defined by the axial diffusivity of the NAWM.

In recent years cognitive dysfunction in MS become the focus point of research. Patients with multiple sclerosis are usually more concerned about their cognitive impairments, than physical disability, and it has a greater influence on their quality of life (54). Earlier results even suggest that there is an accelerated disease progression in the cognitively impaired patients (55). Therefore, the research of the structural background of the cognitive dysfunction cannot be overrated. Recently, BICAMS test, a reliable fast tool for cognitive survey become available, but the structural brain abnormality measured with this test was not systematically investigated.

Not only lesion burden, but lesion localization is a weak predictor of cognitive functions (7). There are several studies showing connection between gray matter atrophy and cognitive functions of MS patients. The volume of the thalamus was found to be associated with information processing speed (56), the attention/executive function and also the verbal memory (57). The volume of the putamen correlated with the information processing speed (SDMT test) (11). Hippocampal atrophy also showed a connection to cognitive dysfunction (58–60). In a multicenter study the atrophy of the hippocampus and the deep GM nuclei was found to be the best predictor of cognitive decline in MS, while the atrophy developed in the WM was associated with the disability of the patients (61).

The thinning of the different regions of the cortex shows connection with various cognitive functions. While the verbal memory performance showed connection with the cortical thinning in the insula, the visual memory performance correlated to parietal atrophy (62), the reduced verbal fluency was associated to the left-sided, while the impaired figural fluency was related to the right-sided cortical thinning in the anterior cingulate region (63). The auditory information processing speed measured with PASAT shows correlation with the thinning of the orbito-frontal cortex (64).

While the above mentioned studies provide evidence about the relationship of cognitive test and certain MRI measures, only limited number of studies investigated the relative contribution of various structural MRI parameters to clinically relevant set of cognitive test. Our results indicate that the three subtest of the BICAMS is related to the pathology of different brain structures.

The most critical contributor to *the visuo-spatial working memory* was the size of the bilateral hippocampi. An earlier study investigating the correlation between various cognitive functions and the components of the thalamic-hippocampal-prefrontal network found no correlation with the visuo-spatial abilities of the patients and the volume of the hippocampus, but the best predictor of the 7/24 Spatial-Recall Task was the uncinate fasciculus connecting the mediotemporal structures to frontal cortex (57). Other studies found the performance of MS patients on visuospatial memory tests correlated with the diffusion parameters of the fornix, the primary hippocampal efferent (65, 66), but not the volume of the hippocampus itself (65). Importantly, the structural abnormality of one structure usually comes along with the other structures connected functionally or structurally (67).

Performance of the *verbal memory* test was best predicted by the volume of the total gray matter and right hippocampus. Interestingly in the above mentioned study of Dineen on a smaller MS group the volume of the hippocampus did not show correlation with the verbal or the visuo-spatial memory scores (65). Kiy and colleagues found association between the consolidation score of the CVLT test and the right temporal horn volume, an indirect measure of hippocampal atrophy (68). A recent study found correlation between physical activity induced hippocampal viscoelasticity and improvement on CVLT-II test in MS patients (69). Global brain atrophy was found to be correlated with the CVLT long delay recall (52). The learning score of the CVLT-II was correlating with the size of the hippocampus, thalamus, caudate and amygdala, but not with the lesion burden or the brain parenchymal fraction in Benedict's study. According to the linear regression analysis the volume of the caudate predicts the verbal learning ability best (70).

The laterality of the hippocampal atrophy correlating with cognitive performance is also interesting. While in case of the BVMT the both hippocampi were similarly predictors of the cognitive function, in case of the CVLT the right hippocampus was significant predictor but not the left. This later is especially interesting since earlier studies showed that left hippocampal volume was related to verbal while the right to the visuospatial memory (71–73).

Recent study of Yu found extensive demyelination-like diffusion alterations in the white matter of MS patients spreading well into the NAWM (74). Most of these microstructural alterations correlated with the performance on the SDMT. Importantly out of the three cognitive test SDMT had the largest area of correlation. Another investigation found SDMT performance be correlated with the gray matter fraction and a diffusion measure of the brain parenchyma (18). Interestingly, the authors used a summary statistics for the diffusion metrics similar to our analysis.

Finally, it is important to consider the methods used in the current analysis. There are two important features of our analysis that needs to be considered. (i) We created a clinically feasible, new whole brain summary measure of the diffusion properties, respecting voxel-wise normal variation of the white matter microstructure. This approach is useful, when a large extent of the diffusion parameters are altered, such as in multiple sclerosis (12).

(ii) The contribution of pattern of diffusion parameters to the clinical and cognitive disability was examined by model-free partial least square analysis. If the predictors show high degree of collinearity (like MRI data), the conventional regression analysis might be misleading.

Partial least squares, besides handling the problem of collinearity, is able to distinguish a pattern of those parameters that best predicts the variable in question.

## Limitations

The main strength of our study is that we have identified the independent contribution of various MRI parameters to the cognitive and clinical disability in MS. Nevertheless, it has to be emphasized that several other quantitative MRI markers were described in multiple sclerosis, such as magnetization transfer imaging, myelin water fraction etc. Including more predictors could possibly give a more through characterization of the dysfunction. Moreover, an important weakness of the approach used in our analysis that we were not considering the spatial distribution of the cortical atrophy. Alternative approaches, such as linked independent component analysis (67) could offer a better characterization of the focal alteration of MRI parameters. It also have to be mentioned that the clinical applicability of theses approach is somewhat limited because of the lengthy data acquisition and processing approaches. However, with advancing MR technology, by establishing the minimum requirements of acquisition parameters (e.g., number of diffusion directions) and improvement of analysis approaches advanced quantitative measures should be available for the clinical practice too.

## Conclusions

In MS there is a complex pathology of focal lesions and diffuse neuro-glial degeneration affecting the gray matter (cortical and subcortical) and also the white matter. These various MRI measurable factors contribute differently to clinical and cognitive disability. Our results indicate that the volumetry of the cortical and subcortical structures and the diffusion measures of the white matter are critical for the understanding the disability progression and these measures should be considered in clinical trials and in the everyday clinical practice.

## Data Availability Statement

All datasets for this study are included in the manuscript and the Supplementary Files.

## Author Contributions

ET, NS, and ZK contributed conception and design of the study. PF, AK, and DS organized the database. BK, KK, and DV performed the statistical analysis. ET wrote the first draft of the manuscript. KB and LV wrote sections of the manuscript. All authors contributed to manuscript revision, read, and approved the submitted version.

### Conflict of Interest Statement

The authors declare that the research was conducted in the absence of any commercial or financial relationships that could be construed as a potential conflict of interest.

## References

[1] Langdon DW. Cognition in multiple sclerosis. Curr Opin Neurol. (2011) 24:244–9. 10.1097/WCO.0b013e328346a43b 21519256

[2] Sandi D Rudisch T Füvesi J Fricska-Nagy Z Huszka H Biernacki T. The Hungarian validation of the brief international cognitive assessment for multiple sclerosis (BICAMS) battery and the correlation of cognitive impairment with fatigue and quality of life. Mult Scler Relat Disord. (2015) 4:499–504. 10.1016/j.msard.2015.07.006 26590654

[3] Walker LA Osman L Berard JA Rees LM Freedman MS MacLean H. Brief International cognitive assessment for multiple sclerosis (BICAMS): canadian contribution to the international validation project. J Neurol Sci. (2016) 362:147–52. 10.1016/j.jns.2016.01.040 26944137

[4] Filippi M Rocca MA Ciccarelli O De Stefano N Evangelou N Kappos L. MRI criteria for the diagnosis of multiple sclerosis: MAGNIMS consensus guidelines. Lancet Neurol. (2016) 15:292–303. 10.1016/S1474-4422(15)00393-2 26822746 PMC4760851

[5] Wattjes MP Rovira À Miller D Yousry TA Sormani MP de Stefano MP. Evidence-based guidelines: MAGNIMS consensus guidelines on the use of MRI in multiple sclerosis–establishing disease prognosis and monitoring patients. Nat Rev Neurol. (2015) 11:597–606. 10.1038/nrneurol.2015.157 26369511

[6] Barkhof F. The clinico-radiological paradox in multiple sclerosis revisited. Curr Opin Neurol. (2002) 15:239–45. 10.1097/00019052-200206000-00003 12045719

[7] Kincses ZT Ropele S Jenkinson M Khalil M Petrovic K Loitfelder M. Lesion probability mapping to explain clinical deficits and cognitive performance in multiple sclerosis. Mult Scler. (2011) 17:681–9. 10.1177/1352458510391342 21177325

[8] Kincses ZT Tóth E Bankó N Veréb D Szabó N Csete G. Grey matter atrophy in patients suffering from multiple sclerosis. Ideggyogy Sz. (2014) 67:293–300. 25518257

[9] De Stefano N Stromillo ML Giorgio A Bartolozzi ML Battaglini M Baldini M. Establishing pathological cut-offs of brain atrophy rates in multiple sclerosis. J Neurol Neurosurg Psychiatry (2015) 87:93–9. 10.1136/jnnp-2014-309903 25904813 PMC4717444

[10] Roosendaal SD Bendfeldt K Vrenken H Polman C H Borgwardt S Radue EW. Grey matter volume in a large cohort of MS patients: relation to MRI parameters and disability. Mult Scler. (2011) 17:1098–106. 10.1177/1352458511404916 21586487

[11] Batista S Zivadinov R Hoogs M Bergsland N Heininen-Brown M Dwyer MG. Basal ganglia, thalamus, and neocortical atrophy predicting slowed cognitive processing in multiple sclerosis. J Neurol. (2012) 259:139–46. 10.1007/s00415-011-6147-1 21720932

[12] Tóth E Szabó N Csete G Király A Faragó P Spisák T. Gray matter atrophy is primarily related to demyelination of lesions in multiple sclerosis: a diffusion tensor imaging MRI study. Front Neuroanat. (2017) 11:23. 10.3389/fnana.2017.00023 28424595 PMC5372801

[13] Hulst HE Steenwijk MD Versteeg A Pouwels PJ Vrenken H Uitdehaag BM. Cognitive impairment in MS: impact of white matter integrity, gray matter volume, and lesions. Neurology (2013) 80:1025–32. 10.1212/WNL.0b013e31828726cc 23468546

[14] Zhang X Zhang F Huang D Wu L Ma L Liu H. Contribution of gray and white matter abnormalities to cognitive impairment in multiple sclerosis. Int J Mol Sci. (2016) 18:46. 10.3390/ijms18010046 28035997 PMC5297681

[15] Filippi M Rocca MA Benedict RH DeLuca J Geurts JJ Rombouts SA. The contribution of MRI in assessing cognitive impairment in multiple sclerosis. Neurology (2010) 75:2121–8. 10.1212/WNL.0b013e318200d768 21135387 PMC3385423

[16] Rocca MA Amato MP De Stefano N Enzinger C Geurts JJ Penner IK. Clinical and imaging assessment of cognitive dysfunction in multiple sclerosis. Lancet Neurol. (2015) 14:302–17. 10.1016/S1474-4422(14)70250-9 25662900

[17] Riccitelli GC Pagani E Rodegher M Colombo B Preziosa P Falini A. Imaging patterns of gray and white matter abnormalities associated with PASAT and SDMT performance in relapsing-remitting multiple sclerosis. Mult Scler. [Epub ahead of print].(2017). 10.1177/1352458517743091. 29173009

[18] Benedict RH Bruce J Dwyer MG Weinstock-Guttman B Tjoa C Tavazzi E. Diffusion-weighted imaging predicts cognitive impairment in multiple sclerosis. Mult Scler. (2007) 13:722–30. 10.1177/1352458507075592 17613599

[19] Hulst HE Gehring K Uitdehaag BM Visser LH Polman CH Barkhof F. Indicators for cognitive performance and subjective cognitive complaints in multiple sclerosis: a role for advanced MRI¿‘ Mult Scler. (2014) 20:1131–4. 10.1177/1352458513513969 24277326

[20] Daams M Steenwijk MD Schoonheim MM Wattjes MP Balk LJ Tewarie PK. Multi-parametric structural magnetic resonance imaging in relation to cognitive dysfunction in long-standing multiple sclerosis. Mult Scler. (2016) 22:608–19. 10.1177/1352458515596598 26209593

[21] Polman CH Reingold SC Edan G Filippi M Hartung HP Kappos L. Diagnostic criteria for multiple sclerosis: 2005 revisions to the “McDonald Criteria”. Ann Neurol. (2005) 58:840–6. 10.1002/ana.20703 16283615

[22] Kurtzke JF. Rating neurologic impairment in multiple sclerosis: an expanded disability status scale (EDSS). Neurology (1983) 33:1444–52. 10.1212/WNL.33.11.1444 6685237

[23] Langdon DW Amato MP Boringa J Brochet B Foley F Fredrikson S. Recommendations for a brief international cognitive assessment for multiple sclerosis (BICAMS). Mult Scler. (2012) 18:891–8. 10.1177/1352458511431076 22190573 PMC3546642

[24] Smith SM Zhang Y Jenkinson M Chen J Matthews PM Federico A. Accurate, robust, and automated longitudinal and cross-sectional brain change analysis. Neuroimage (2002) 17:479–89. 10.1006/nimg.2002.1040 12482100

[25] Smith SM Jenkinson M Woolrich MW Beckmann CF Behrens TE Johansen-Berg H. Advances in functional and structural MR image analysis and implementation as FSL. Neuroimage (2004) 23 (Suppl. 1):S208–19. 10.1016/j.neuroimage.2004.07.051 15501092

[26] Jenkinson M Beckmann CF Behrens TE Woolrich MW Smith SM. Fsl. Neuroimage (2012) 62:782–90. 10.1016/j.neuroimage.2011.09.015 21979382

[27] Zhang Y Brady M Smith S. Segmentation of brain MR images through a hidden Markov random field model and the expectation-maximization algorithm. IEEE Trans Med Imaging (2001) 20:45–57. 10.1109/42.906424 11293691

[28] Patenaude B Smith SM Kennedy DN Jenkinson M. A Bayesian model of shape and appearance for subcortical brain segmentation. Neuroimage (2011) 56:907–22. 10.1016/j.neuroimage.2011.02.046 21352927 PMC3417233

[29] Zarei M Patenaude B Damoiseaux J Morgese C Smith S Matthews PM. Combining shape and connectivity analysis: an MRI study of thalamic degeneration in Alzheimer's disease. Neuroimage (2010) 49:1–8. 10.1016/j.neuroimage.2009.09.001 19744568

[30] Abdi H Williams LJ. Partial least squares methods: partial least squares correlation and partial least square regression. Methods Mol Biol. (2013) 930:549–79. 10.1007/978-1-62703-059-5_23 23086857

[31] Wold S Johansson E Cocchi M (1994). PLS - Partial Least Squares Projections to Latent Structures. 3D QSAR in drug design volume 1. In: Kubinyi H, editor. Theory Methods and Applications. Leiden: ESCOM Science Publishers 523–50.

[32] De Stefano N Matthews PM Filippi M Agosta F De Luca M Bartolozzi ML. Evidence of early cortical atrophy in MS: relevance to white matter changes and disability. Neurology (2003) 60:1157–62. 10.1212/01.WNL.0000055926.69643.03 12682324

[33] Amato MP Bartolozzi ML Zipoli V Portaccio E Mortilla M Guidi L. Neocortical volume decrease in relapsing-remitting MS patients with mild cognitive impairment. Neurology (2004) 63:89–93. 10.1212/01.WNL.0000129544.79539.D5 15249616

[34] Sanfilipo MP Benedict RH Sharma J Weinstock-Guttman B Bakshi R. The relationship between whole brain volume and disability in multiple sclerosis: a comparison of normalized gray vs. white matter with misclassification correction. Neuroimage (2005) 26:1068–77. 10.1016/j.neuroimage.2005.03.008 15961046

[35] Tedeschi G Lavorgna L Russo P Prinster A Dinacci D Savettier G. Brain atrophy and lesion load in a large population of patients with multiple sclerosis. Neurology (2005) 65:280–5. 10.1212/01.wnl.0000168837.87351.1f 16043800

[36] Fisniku LK Chard DT Jackson JS Anderson VM Altmann DR Miszkiel KA. Gray matter atrophy is related to long-term disability in multiple sclerosis. Ann Neurol. (2008) 64:247–54. 10.1002/ana.21423 18570297

[37] Morgen K Sammer G Courtney SM Wolters T Melchior H Blecker CR. Evidence for a direct association between cortical atrophy and cognitive impairment in relapsing-remitting MS. Neuroimage (2006) 30:891–8. 10.1016/j.neuroimage.2005.10.032 16360321

[38] Prinster A Quarantelli M Orefice G Lanzillo R Brunetti A Mollica C. Grey matter loss in relapsing-remitting multiple sclerosis: a voxel-based morphometry study. Neuroimage (2006) 29:859–67. 10.1016/j.neuroimage.2005.08.034 16203159

[39] Khaleeli Z Cercignani M Audoin B Ciccarelli O Miller DH Thompson AJ. Localized grey matter damage in early primary progressive multiple sclerosis contributes to disability. Neuroimage (2007) 37:253–61. 10.1016/j.neuroimage.2007.04.056 17566765

[40] Sastre-Garriga J Arevalo MJ Renom M Alonso J González I Galán I. Brain volumetry counterparts of cognitive impairment in patients with multiple sclerosis. J Neurol Sci. (2009) 282:120–4. 10.1016/j.jns.2008.12.019 19157420

[41] Ceccarelli A Jackson JS Tauhid S Arora A Gorky J Dell'Oglio E. The impact of lesion in-painting and registration methods on voxel-based morphometry in detecting regional cerebral gray matter atrophy in multiple sclerosis. AJNR Am J Neuroradiol. (2012) 33:1579–85. 10.3174/ajnr.A3083 22460341 PMC3425668

[42] Cerasa A Valentino P Chiriaco C Pirritano D Nisticò R Gioia CM. MR imaging and cognitive correlates of relapsing-remitting multiple sclerosis patients with cerebellar symptoms. J Neurol. (2013) 260:1358–66. 10.1007/s00415-012-6805-y 23271221

[43] Griffin CM Chard DT Ciccarelli O Kapoor B Barker GJ Thompson A. I.. Diffusion tensor imaging in early relapsing-remitting multiple sclerosis. Mult Scler. (2001) 7:290–7. 10.1177/135245850100700504 11724444

[44] Filippi M Cercignani M Inglese M Horsfield MA Comi G. Diffusion tensor magnetic resonance imaging in multiple sclerosis. Neurology (2001) 56:304–11. 10.1212/WNL.56.3.304 11171893

[45] Hasan KM Gupta RK Santos RM Wolinsky JS Narayana PA. Diffusion tensor fractional anisotropy of the normal-appearing seven segments of the corpus callosum in healthy adults and relapsing-remitting multiple sclerosis patients. J Magn Reson Imaging (2005) 21:735–43. 10.1002/jmri.20296 15906348

[46] Wilson M Tench CR Morgan PS Blumhardt LD. Pyramidal tract mapping by diffusion tensor magnetic resonance imaging in multiple sclerosis: improving correlations with disability. J Neurol Neurosurg Psychiatry (2003) 74:203–7. 10.1136/jnnp.74.2.203 12531950 PMC1738288

[47] Onu M Roceanu A Sboto-Frankenstein U Bendic R Tarta E Preoteasa F. Diffusion abnormality maps in demyelinating disease: correlations with clinical scores. Eur J Radiol. (2012) 81:e386–91. 10.1016/j.ejrad.2011.12.014 22257426

[48] Ciccarelli O Werring DJ Wheeler-Kingshott CA Barker GJ Parker GJ Thompson AJ. Investigation of MS normal-appearing brain using diffusion tensor MRI with clinical correlations. Neurology (2001) 56:926–33. 10.1212/WNL.56.7.926 11294931

[49] Pierpaoli C Barnett A Pajevic S Chen R Penix LR Virta A. Water diffusion changes in Wallerian degeneration and their dependence on white matter architecture. Neuroimage (2001) 13(6 Pt 1):1174–85. 10.1006/nimg.2001.0765S1053811901907657 11352623

[50] Sun SW Liang HF Trinkaus K Cross AH Armstrong RC Song SK. Noninvasive detection of cuprizone induced axonal damage and demyelination in the mouse corpus callosum. Magn Reson Med. (2006) 55:302–8. 10.1002/mrm.20774 16408263

[51] Kim JH Budde MD Liang HF Klein RS Russell JH Cross AH. Detecting axon damage in spinal cord from a mouse model of multiple sclerosis. Neurobiol Dis. (2006) 21:626–32. 10.1016/j.nbd.2005.09.009 16298135

[52] Sun SW Liang HF Cross AH Song SK. Evolving Wallerian degeneration after transient retinal ischemia in mice characterized by diffusion tensor imaging. NeuroImage (2008) 40:1–10. 10.1016/j.neuroimage.2007.11.049 18187343 PMC2276530

[53] Zhang J Jones M DeBoy CA Reich DS Farrell JA Hoffman PN. Diffusion tensor magnetic resonance imaging of Wallerian degeneration in rat spinal cord after dorsal root axotomy. J Neurosci. (2009) 29:3160–71. 10.1523/JNEUROSCI.3941-08.2009 19279253 PMC2683764

[54] Fricska-Nagy Z Fuvesi J Rózsa C Komoly S Jakab G Csépány T. The effects of fatigue, depression and the level of disability on the health-related quality of life of glatiramer acetate-treated relapsing-remitting patients with multiple sclerosis in Hungary. Mult Scler Relat Disord. (2016) 7:26–32. 10.1016/j.msard.2016.02.006 27237753

[55] Sander C Eling P Hanken K Klein J Kastrup A Hildebrandt H. The impact of MS-related cognitive fatigue on future brain parenchymal loss and relapse: a 17-month follow-up study. Front Neurol. (2016) 7:155. 10.3389/fneur.2016.00155 27708613 PMC5030297

[56] Koini M Filippi M Rocca MA Yousry T Ciccarelli O Tedeschi G. Correlates of executive functions in multiple sclerosis based on structural and functional MR imaging: insights from a multicenter study. Radiology (2016) 280:869–79. 10.1148/radiol.2016151809 27002420

[57] Kern KC Gold SM Lee B Montag M Horsfall J O'Connor MF. Thalamic-hippocampal-prefrontal disruption in relapsing-remitting multiple sclerosis. Neuroimage Clin. (2015) 8:440–7. 10.1016/j.nicl.2014.12.015 26106524 PMC4473119

[58] Hulst HE Schoonheim MM Van Geest Q Uitdehaag BM Barkhof F Geurts JJ. Memory impairment in multiple sclerosis: relevance of hippocampal activation and hippocampal connectivity. Mult Scler. (2015) 21:1705–12. 10.1177/1352458514567727 25680986

[59] Sacco R Bisecco A Corbo D Della Corte M d'Ambrosio A Docimo R. Cognitive impairment and memory disorders in relapsing-remitting multiple sclerosis: the role of white matter, gray matter and hippocampus. J Neurol. (2015) 262:1691–7. 10.1007/s00415-015-7763-y 25957638

[60] Rocca MA Morelli ME Amato MP Moiola L Ghezzi A Veggiotti P. Regional hippocampal involvement and cognitive impairment in pediatric multiple sclerosis. Mult Scler. (2016) 22:628–40. 10.1177/1352458515598569 26286701

[61] Damjanovic D Valsasina P Rocca MA Stromillo ML Gallo A Enzinger C. Hippocampal and deep gray matter nuclei atrophy is relevant for explaining cognitive impairment in MS: a multicenter study. AJNR Am J Neuroradiol. (2017) 38:18–24. 10.3174/ajnr.A4952 27686487 PMC7963669

[62] Tillema JM Hulst HE Rocca MA Vrenken H Steenwijk MD Damjanovic D. Regional cortical thinning in multiple sclerosis and its relation with cognitive impairment: a multicenter study. Mult Scler. (2016) 22:901–9. 10.1177/1352458515607650 26432859

[63] Geisseler O Pflugshaupt T Bezzola L Reuter K Weller D Schuknecht B. Cortical thinning in the anterior cingulate cortex predicts multiple sclerosis patients' fluency performance in a lateralised manner. Neuroimage Clin. (2016) 10:89–95. 10.1016/j.nicl.2015.11.008 26759784 PMC4683425

[64] Sbardella E Petsas N Tona F Prosperini L Raz E Pace G. Assessing the correlation between grey and white matter damage with motor and cognitive impairment in multiple sclerosis patients. PLoS ONE (2013) 8:e63250. 10.1371/journal.pone.0063250 23696802 PMC3655958

[65] Dineen RA Bradshaw CM Constantinescu CS Auer DP. Extra-hippocampal subcortical limbic involvement predicts episodic recall performance in multiple sclerosis. PLoS ONE (2012) 7:e44942. 10.1371/journal.pone.0044942 23056187 PMC3466267

[66] Koenig KA Sakaie KE Lowe MJ Lin J Stone L Bermel RA. High spatial and angular resolution diffusion-weighted imaging reveals forniceal damage related to memory impairment. Magn Reson Imaging (2013) 31:695–9. 10.1016/j.mri.2012.10.030 23295147 PMC3648592

[67] Kincses ZT Horinek D Szabo N Toth E Csete G Stepan-Buksakowska I. The pattern of diffusion parameter changes in Alzheimer's disease, identified by means of linked independent component analysis. J Alzheimers Dis. (2013) 36:119–28. 10.3233/JAD-122431 23542867

[68] Kiy G Lehmann P Hahn HK Eling P Kastrup A Hildebrandt H. Decreased hippocampal volume, indirectly measured, is associated with depressive symptoms and consolidation deficits in multiple sclerosis. Mult Scler. (2011) 17:1088–97. 10.1177/1352458511403530 21546523

[69] Sandroff BM Johnson CL Motl RW. Exercise training effects on memory and hippocampal viscoelasticity in multiple sclerosis: a novel application of magnetic resonance elastography. Neuroradiology (2017) 59:61–7. 10.1007/s00234-016-1767-x 27889837

[70] Benedict RH Ramasamy D Munschauer F Weinstock-Guttman B Zivadinov R. Memory impairment in multiple sclerosis, correlation with deep grey matter and mesial temporal atrophy. J Neurol Neurosurg Psychiatry (2009) 80:201–6. 10.1136/jnnp.2008.148403 18829629

[71] Shi F Liu B Zhou Y Yu C Jiang T. Hippocampal volume and asymmetry in mild cognitive impairment and Alzheimer's disease: meta-analyses of MRI studies. Hippocampus (2009) 19:1055–64. 10.1002/hipo.20573 19309039

[72] Travis SG Huang Y Fujiwara E Radomski A Olsen F Carter R. High field structural MRI reveals specific episodic memory correlates in the subfields of the hippocampus. Neuropsychologia (2014) 53:233–45. 10.1016/j.neuropsychologia.2013.11.016 24296251

[73] Ezzati A Katz MJ Zammit AR Lipton ML Zimmerman ME Sliwinski MJ. Differential association of left and right hippocampal volumes with verbal episodic and spatial memory in older adults. Neuropsychologia (2016) 93(Pt B):380–5. 10.1016/j.neuropsychologia.2016.08.016 27542320 PMC5154822

[74] Yu HJ Christodoulou C Bhise V Greenblatt D Patel Y Serafin D. Multiple white matter tract abnormalities underlie cognitive impairment in RRMS. Neuroimage (2012) 59:3713–22. 10.1016/j.neuroimage.2011.10.053 22062194

